# Comparison of angiography-guided vs. intra-vascular imaging-guiding percutaneous coronary intervention of acute myocardial infarction: a real world clinical practice

**DOI:** 10.3389/fcvm.2024.1421025

**Published:** 2024-08-29

**Authors:** Ting-Yu Lin, Ying-Ying Chen, Shao-Sung Huang, Cheng-Hsueh Wu, Li-Wei Chen, Yu-Lun Cheng, William K. Hau, Chien-Hung Hsueh, Ming-Ju Chuang, Wei-Chieh Huang, Tse-Min Lu

**Affiliations:** ^1^ Cardiovascular Medical Center, Kaohsiung Veterans General Hospital, Kaohsiung, Taiwan; ^2^ Division of Cardiology, Department of Internal Medicine, Taipei Veterans General Hospital, Taipei, Taiwan; ^3^ Division of Nephrology, Department of Internal Medicine, MacKay Memorial Hospital, Taipei, Taiwan; ^4^ Department of Internal Medicine, School of Medicine, College of Medicine, National Yang Ming Chiao Tung University, Taipei, Taiwan; ^5^ Department of Health Care Center, Taipei Veterans General Hospital, Taipei, Taiwan; ^6^ Division of Cardiology, Department of Internal Medicine, Taipei Medical University Hospital, Taipei, Taiwan; ^7^ Division of Cardiology, Department of Internal Medicine, New Taipei City Hospital, New Taipei City, Taiwan; ^8^ Department of Medicine and Therapeutics, The Chinese University of Hong Kong, Hong Kong SAR, China; ^9^ Division of Cardiology, Department of Internal Medicine, Taichung Veterans General Hospital, Taichung, Taiwan; ^10^ Department of Biomedical Engineering, National Taiwan University, Taipei, Taiwan

**Keywords:** acute myocardial infarction (AMI), percutaneous coronary intervention (PCI), optical coherence tomography (OCT), intravascular ultrasound (IVUS), intravascular image, MACE

## Abstract

**Background:**

The role of routine intravascular imaging in percutaneous coronary intervention (PCI) for acute myocardial infarction (AMI) remains unclear. This study evaluated the clinical outcomes of PCI guided by different imaging modalities in AMI patients.

**Materials and methods:**

Data from AMI patients who had undergone PCI between 2012 and 2022 were analyzed. The mean follow-up was 12.9 ± 1.73 months. The imaging modality-either intravascular ultrasound (IVUS), optical coherence tomography (OCT), or angiography alone-was selected at the operator's discretion. The primary endpoint was major adverse cardiac events (MACEs), including cardiovascular (CV) death, myocardial infarction (MI), target vessel revascularization.

**Results:**

Of the 1,304 PCIs performed, 47.5% (*n* = 620) were guided by angiography alone, 37.0% (*n* = 483) by IVUS, and 15.4% (*n* = 201) by OCT. PCI guided by intravascular imaging modalities was associated with lower 1-year rates of MI (1.3%, *P* = 0.001) and MACE (5.2%, *P* = 0.036). OCT-guided PCI was linked to lower rates of 1-year CV death (IVUS vs. OCT: 6.2% vs. 1.5%, *P* = 0.016) and MACE (IVUS vs. OCT: 6.4% vs. 2.5%, *P* = 0.032). Intravascular imaging modalities and diabetes were identified as predictors of better and worse 1-year MACE outcomes, respectively.

**Conclusion:**

PCI guided by intravascular imaging modalities resulted in improved 1-year clinical outcomes compared to angiography-guided PCI alone in AMI patients. OCT-guided PCI was associated with lower 1-year MACE rates compared to IVUS-guided PCI. Therefore, intravascular imaging should be recommended for PCI in AMI, with OCT being particularly considered when appropriate.

## Introduction

Emergent reperfusion of the ischemic myocardium represents the most significant advancement in treating acute myocardial infarction (AMI) over the past three decades. Studies have demonstrated that adequate reperfusion in AMI patients leads to reduced infarct size, lower early death rates, preserved left ventricular function, and improved survival (1, 2). The culprit lesion in AMI often arises from acute occlusion of a major coronary artery due to a large thrombus, vessel spasm, or acute coronary dissection. Identifying the “true” culprit lesion using angiography alone is challenging, as it may be located proximal or distal to the angiographically identified site (3). This difficulty poses a challenge for interventional cardiologists in selecting the appropriate stent placement zone and in choosing the correct stent size and length for AMI patients undergoing PCI. Both Intravascular ultrasound (IVUS) and optical coherence tomography (OCT) imaging provide tomographic cross-sectional imaging of the vessel wall, offering valuable morphological information regarding the coronary lesion, aiding in stent size selection, optimizing stent expansion based on vessel diameter, and detecting incomplete apposition, longitudinal stent deformation and/or edge dissection (4–10). A large observational cohort study from the Pan-London PCI registry documented that OCT-guided PCI in patients with stable coronary artery disease was associated with improved procedural outcomes, in-hospital events, and long-term survival compared to those associated with standard angiography-guided PCI alone (11). Additionally, a few non-inferiority trials have shown no difference in cardiovascular outcomes between OCT-guided PCI and IVUS-guided PCI in elective PCI patients (12–14). Recently in a 2024 meta-analysis comparing intravascular imaging-guided and angiography-guided PCI, it was found that using intravascular imaging (OCT or intravascular ultrasound) improves PCI safety and effectiveness (15). This includes reduced risks of death, myocardial infarction, stent thrombosis, and repeat revascularization compared to angiography alone. Notably, outcomes were similar between OCT-guided and intravascular ultrasound-guided PCI. In another multicenter randomized controlled trial, IVUS-guided PCI was found to significantly reduce 1-year rates of target vessel failure in ACS patients compared to angiography-guided PCI, due to fewer MIs and revascularizations (16). However, the benefits of routine incorporating invasive imaging modalities (IVUS or OCT) into AMI PCI and the comparison between these modalities remain controversial in real-world clinical practice (17–19). Therefore, this study aimed to compare clinical outcomes between angiography alone and invasive imaging modalities (IVUS and OCT)-guided PCI in AMI patients.

## Methods

### Study population and enrollment

**Figure 1:**
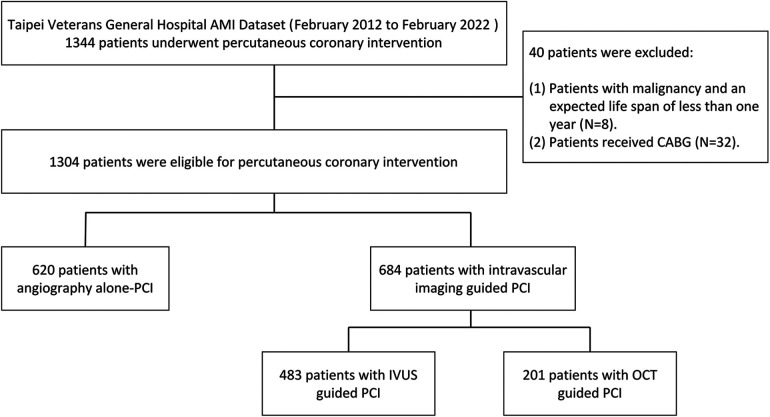
The flow of study. CABG: Coronary artery bypass graft surgery.

This retrospective registry comprised consecutive AMI patients who had undergone PCI from February 2012 to February 2022 (Figure 1). Chronic kidney disease (CKD) was defined as eGFR < 60 ml/min/1.73 m2. AMI was characterized by the presence of significant new Q waves in at least two electrocardiography leads or symptoms compatible with MI, accompanied by an increase in cTn above the 99th percentile upper reference limit (20). Data on baseline and procedural characteristics, medical history, clinical examination, operation records, and clinical outcomes were collected through a medical chart review. AMI PCI was conducted following the standard practices of our hospital. The procedures were performed using either the radial or femoral approach. All patients received dual antiplatelet therapy before the procedure, and the activated clotting time was maintained between 250 and 300 s throughout the procedure. The administration of glycoprotein IIb/IIIa receptor antagonists and/or manual thrombus aspiration was at the operator's discretion. Patients were categorized based on the imaging modalities used to evaluate lesion characteristics, which included angiography alone, angiography plus IVUS, and angiography plus OCT. The selection of the invasive imaging modality was at the operator's discretion. Although there are no strict written rules at our hospital, the indications and systematic criteria were based on the global current consensus for pre- and post-stenting goals: (1) selecting suitable patients, (2) pre-stenting balloon sizing, (3) stent sizing, (4) post-stenting balloon sizing, (5) ensuring complete apposition and adequate expansion (no underexpansion or malapposition), and (6) avoiding edge dissection (21). The study protocol received approval from the institutional review board at Taipei Veterans General Hospital.

### Imaging modalities (IVUS and OCT)

Our hospital is a medical center with a high volume of intravascular procedures. Intravascular imaging is routinely performed in nearly all PCIs, with an 80% penetration rate in stable coronary artery disease. In AMI PCI, invasive imaging (IVUS and OCT) may be performed after the restoration of TIMI flow in the target vessel, either after thrombectomy or the use of small balloon inflation. In this study, operators carefully reviewed the IVUS and OCT imaging results and chose the landing zone and the stent size and length. After stent implantation, IVUS or OCT pullback was performed again to identify any suboptimal results, such as stent under-expansion or malposition.

### Clinical follow-up and outcomes

In-hospital complications were documented at the time of discharge. Clinical outcomes, including death, myocardial infarction (MI), and target vessel revascularization (TVR), were recorded 12 months post-discharge. MACE, a composite endpoint, encompasses cardiovascular (CV) death, MI, and TVR. CV death is classified as any death definitively caused by CV issues or any death not explicitly attributed to non-CV causes. Non-fatal MI is characterized by significant new Q waves in at least two electrocardiography leads or symptoms indicative of MI, accompanied by an elevation in cTn above the 99th percentile upper reference limit (20). TVR is the clinically driven repeat revascularization during follow-up due to restenosis, either within the target lesion or the same epicardial coronary artery. Confirmation of all cardiac events was obtained through a review of patient medical records via a dedicated electronic system, which recorded patient events, hospitalizations, and outpatient clinic follow-up details.

### Statistical analysis

Continuous and categorical variables were represented as means ± standard deviation and numbers with percentages, respectively. We used Chi-square or Fisher's exact test for categorical variables and Student's *t*-test, one-way ANOVA, or Kruskal–Wallis test for continuous variables to compare baseline characteristics between groups. We conducted PSM at a 1:1 ratio to ensure robust matching of patients. The matching criteria included age, gender, diabetes mellitus (DM), chronic kidney disease (CKD)/end-stage renal disease (ESRD), and congestive heart failure (CHF). These variables were chosen to reduce bias from confounding variables by indication. To evaluate the effectiveness of the matching process, we calculated standardized mean differences (SMDs) before and after matching, and the SMDs below the threshold of 0.1 for all included variables. Cox regression model is performed to examine the association of clinical outcomes within 1 year follow-up period. The hazard ratio (HR) and 95% CI are calculated and *p* value of <0.05 is considered to be statistically significant. The Kaplan–Meier (KM) method was used to calculate the cumulative survival rate for different imaging modalities. All tests were two-tailed, and *P* values ≤0.05 were considered statistically significant.

## Results

### Demographic and procedural characteristics

During the study period, 1,304 consecutive AMI patients who had undergone PCI were enrolled. Of these, 47.5% (*n* = 620) underwent angiography-guided PCI, 37.0% (*n* = 483) underwent IVUS-guided PCI, and 15.4% (*n* = 201) underwent OCT-guided PCI. We classified the patients into two groups: those who received angiography-guided PCI alone and those who received intravascular imaging-guided PCI. The baseline and procedural characteristics of the enrolled patients are discussed in Table 1. Patients in the intravascular imaging-guided PCI group had more stents used (2.9 ± 1.4, *P* < 0.001) and greater stent lengths (23.2 ± 9.0, *P* < 0.001). The propensity score matching (PSM) for assessing the impact of imaging modalities is discussed in Table 2. The patients in the intravascular imaging-guided PCI group still had greater stent lengths (22.1 ± 8.3, *P* < 0.001).

**Table 1:** Demographic & procedural data of patients with acute myocardial infarction.

Variables	Angiography alone	Intra-vascular imaging	*P*-value
	*N* = 620	*N* = 684	
Baseline characteristics			
Male	514 (82.9%)	564 (82.5%)	0.961
Age	62.3 ± 11.8	62.5 ± 10.8	0.971
Hypertension	316 (51.0%)	338 (49.4%)	0.810
Diabetes	143 (23.1%)	156 (22.8%)	0.890
Hyperlipidemia	248 (40.0%)	207 (30.3%)	0.531
CKD/ESRD	176 (28.4%)	157 (23.0%)	0.734
Smoker	221 (35.6%)	241 (35.2%)	0.946
Clinical presentation			
NSTEMI	385 (62.1%)	402 (58.8%)	0.892
STEMI	235 (37.9%)	282 (41.2%)	0.811
Cardiac history			
History of MI (>3M)	41 (6.6%)	39 (5.7%)	0.767
History of CHF	46 (7.4%)	38 (5.6%)	0.453
Prior PCI	40 (6.5%)	38 (5.7%)	0.763
LVEF (%)	56.3 ± 10.3	51.6 ± 11.3	0.246
Number of vessel disease			0.716
Single	310 (50.0%)	327 (47.8%)	
Double	217 (35.0%)	250 (36.5%)	
Triple	93 (15.0%)	107 (15.6%)	
Targeted vessel			
LM	10 (1.6%)	38 (5.6%)	0.001
LAD	370 (59.7%)	428 (62.6%)	0.484
LCX	143 (23.1%)	128 (18.7%)	0.186
RCA	270 (43.5%)	306 (44.7%)	0.889
Procedural characteristics			
Number of stents	1.6 ± 0.9	2.9 ± 1.4	<0.001
Stent length	18.8 ± 7.2	23.2 ± 9.0	<0.001
DES	467 (75.3%)	536 (78.4%)	0.832
BMS	153 (24.7%)	148 (21.6%)	0.732
Laboratory data			
T. Cholesterol (mg/dl)	153.6 ± 37.5	158.3 ± 44.2	0.366
LDL-C (mg/dl)	90.4 ± 34.0	93.9 ± 35.7	0.437
eGFR	62.4 ± 24.3	58.4 ± 26.6	0.768
HbA1c	6.71 ± 1.58	6.87 ± 1.27	0.761

BMS, bare metal stent; CHF, congestive heart failure; CKD/ESRD, chronic kidney disease/end stage renal disease; DES, drug eluting stent; eGFR, estimated glomerular filtration rate; HbA1c, hemoglobin A1C; LAD, left anterior descending coronary artery; LCX, left circumflex coronary artery; LDL-C, low density lipoprotein cholesterol; LM, left main coronary artery; LVEF, left ventricular ejection fraction; MI, myocardial infarction; NSTEMI, non ST segment elevation myocardial infarction; PCI, percutaneous coronary intervention; RCA, right coronary artery; STEMI, ST segment elevation myocardial infarction.

**Table 2:** Propensity score matching of the impact of imaging modalities .

Variables	Angiography alone	Intra-vascular imaging	*P*-value
	*N* = 598	*N* = 598	
Characteristics			
Male	493 (82.4%)	486 (81.3%)	0.971
Age	62.8 ± 11.4	62.1 ± 9.7	0.579
Hypertension	287 (48.0%)	257 (43.0%)	0.442
Diabetes	102 (17.1%)	116 (19.4%)	0.621
Hyperlipidaemia	245 (40.9%)	180 (30.1%)	0.421
CKD/ESRD	163 (27.3%)	148 (24.7%)	0.784
Smoker	198 (33.1%)	204 (34.1%)	0.836
Clinical presentation			
NSTEMI	365 (61.0%)	346 (57.9%)	0.862
STEMI	233 (39.0%)	252 (42.1%)	0.801
Cardiac history			
History of MI (>3M)	30 (5.0%)	35 (5.9%)	0.701
History of CHF	42 (7.0%)	37 (6.2%)	0.643
Prior PCI	32 (5.4%)	33 (5.5%)	0.863
LVEF (%)	56.1 ± 9.5	55.6 ± 10.4	0.946
No. vessel disease			0.702
Single	299 (50.0%)	279 (46.7%)	
Double	208 (34.8%)	235 (39.2%)	
Triple	91 (15.2%)	84 (14.0%)	
Target vessel			
LM	7 (1.2%)	14 (2.3%)	0.413
LAD	349 (58.4%)	364 (60.9%)	0.697
LCX	135 (22.6%)	103 (17.2%)	0.216
RCA	267 (44.6%)	301 (50.3%)	0.371
No. of stents used	1.6 ± 1.0	2.6 ± 1.1	0.261
Stent length	18.2 ± 6.1	22.1 ± 8.3	<0.001
DES	450 (75.2%)	458 (76.6%)	0.912
BMS	148 (24.7%)	140 (23.4%)	0.892
Laboratory data			
T. Cholesterol (mg/dl)	155.1 ± 32.5	158.1 ± 41.2	0.322
LDL-C (mg/dl)	90.1 ± 34.1	93.2 ± 35.2	0.431
eGFR	62.3 ± 24.1	58.1 ± 26.3	0.760
HbA1c	6.70 ± 1.49	6.84 ± 1.22	0.760

BMS, bare metal stent; CHF, congestive heart failure; CKD/ESRD, chronic kidney disease/end stage renal disease; DES, drug eluting stent; eGFR, estimated glomerular filtration rate; HbA1c, hemoglobin A1C; LAD, left anterior descending coronary artery; LCX, Left circumflex coronary artery; LDL-C, low density lipoprotein cholesterol; LM, left main coronary artery; LVEF, left ventricular ejection fraction; MI, myocardial infarction; NSTEMI, non ST segment elevation myocardial infarction; PCI, percutaneous coronary intervention; RCA, right coronary artery; STEMI, ST segment elevation myocardial infarction.

In the subgroup analysis, patients undergoing intravascular imaging-guided PCI were categorized into OCT-guided and IVUS-guided PCI groups (Table 3). Those in the OCT-guided PCI group were older (63.5 ± 11.3, *P* = 0.001) and exhibited a greater incidence of multi-vessel disease (*P* = 0.001). The PSM for the subgroup analysis is shown in Table 4. After matching, no significant differences were observed between the two groups, except for a higher prevalence of LM disease in the OCT-guided PCI group (*P* = 0.032).

**Table 3:** Characteristics of enrolled acute myocardial infarction patients in IVUS guided and OCT guided group.

Variables	IVUS *N* = 483	OCT *N* = 201	*P*-value
Baseline Characteristics			
Male	396 (82.0%)	168 (83.5%)	0.866
Age	60.5 ± 10.5	63.5 ± 11.3	0.001
Hypertension	245 (50.7%)	93 (46.3%)	0.782
Diabetes	103 (21.3%)	53 (26.3%)	0.573
Hyperlipidemia	135 (28.0%)	72 (35.8%)	0.239
CKD/ESRD	110 (22.7%)	47 (23.4%)	0.722
Smoker	177 (36.6%)	64 (31.8%)	0.261
Clinical presentation			
NSTEMI	284 (58.8%)	118 (58.7%)	0.922
STEMI	200 (41.4%)	82 (40.7%)	0.902
Cardiac History			
History of MI (>3M)	24 (5.0%)	15 (7.5%)	0.207
History of CHF	24 (5.0%)	14 (7.0%)	0.424
Prior PCI	25 (5.2%)	13 (6.5%)	0.653
LVEF (%)	52.5 ± 10.1	50.5 ± 9.3	0.592
Number of Vessel disease			0.001
Single	251 (52.0%)	76 (37.8%)	
Double	168 (34.8%)	82 (40.8%)	
Triple	64 (13.3%)	43 (21.4%)	
Targeted Vessel			
LM	18 (3.7%)	20 (10.0%)	0.013
LAD	308 (63.8%)	120 (59.7%)	0.373
LCX	71 (14.7%)	57 (28.4%)	<0.001
RCA	215 (44.5%)	91 (45.3%)	0.872
Procedural characteristics			
Number of stents	2.7 ± 1.1	3.1 ± 1.2	0.382
Stent length	22.6 ± 6.2	24.2 ± 7.1	0.362
DES	385 (79.7%)	151 (75.1%)	0.862
BMS	98 (20.3%)	50 (24.9%)	
Laboratory data			
T. Cholesterol (mg/dl)	157.1 ± 42.5	159.1 ± 41.2	0.322
LDL-C (mg/dl)	92.1 ± 32.1	95.2 ± 32.2	0.431
eGFR	59.1 ± 22.1	58.1 ± 23.3	0.760
HbA1c	6.80 ± 1.61	6.88 ± 1.28	0.769

BMS, bare metal stent; CHF, congestive heart failure; CKD/ESRD, chronic kidney disease/end stage renal disease; DES, drug eluting stent; eGFR, estimated glomerular filtration rate; HbA1c, hemoglobin A1C; LAD, left anterior descending coronary artery; LCX, left circumflex coronary artery; LDL-C, low density lipoprotein cholesterol; LM, left main coronary artery; LVEF, left ventricular ejection fraction; MI, myocardial infarction; NSTEMI, non ST segment elevation myocardial infarction; PCI, percutaneous coronary intervention; RCA, right coronary artery; STEMI, ST segment elevation myocardial infarction.

**Table 4:** Propensity score matching of the impact of different imaging modalities.

Variables	IVUS	OCT	*P*-value
	*N* = 190	*N* = 190	
Characteristics			
Male	157 (82.6%)	156 (82.1%)	0.864
Age	61.9 ± 11.4	62.6 ± 10.5	0.631
Hypertension	80 (42.1%)	81 (42.6%)	0.999
Diabetes	45 (23.7%)	42 (22.1%)	0.757
Hyperlipidaemia	61 (32.2%)	56 (29.5%)	0.673
CKD/ESRD	40 (21.1%)	44 (23.2%)	0.763
Smoker	72 (37.9%)	60 (31.6%)	0.489
Clinical presentation			
NSTEMI	109 (57.4%)	111 (58.4%)	0.911
STEMI	81 (42.6%)	79 (41.6%)	0.908
Cardiac History			
History of MI (>3M)	7 (3.7%)	10 (5.3%)	0.451
History of CHF	11 (5.8%)	12 (6.3%)	0.824
Prior PCI	11 (5.8%)	9 (4.7%)	0.683
LVEF (%)	51.9 ± 10.4	52.6 ± 12.5	0.566
No. Vessel disease			0.379
Single	83 (43.7%)	70 (36.8%)	
Double	70 (36.8%)	75 (39.9%)	
Triple	37 (19.5%)	45 (23.7%)	
Target Vessel			
LM	6 (3.2%)	16 (8.4%)	0.032
LAD	115 (60.5%)	111 (58.4%)	0.511
LCX	27 (14.2%)	49 (25.8%)	0.332
RCA	76 (40.0%)	74 (38.9%)	0.894
Procedural characteristics			
No. of stents used	2.4 ± 0.9	2.8 ± 1.2	0.312
Stent length	22.0 ± 8.8	23.7 ± 9.3	0.370
DES	145 (76.3%)	139 (73.2%)	0.769
BMS	45 (23.7%)	51 (26.8%)	
Laboratory data			
T. Cholesterol (mg/dl)	157.3 ± 40.5	159.0 ± 40.1	0.302
LDL-C (mg/dl)	91.1 ± 31.1	96.2 ± 31.2	0.231
eGFR	58.8 ± 21.1	58.3 ± 22.3	0.802
HbA1c	6.72 ± 1.6	6.87 ± 1.3	0.779

BMS, bare metal stent; CHF, congestive heart failure; CKD/ESRD, chronic kidney disease/end stage renal disease; DES, drug eluting stent; eGFR, estimated glomerular filtration rate; HbA1c, hemoglobin A1C; LAD, left anterior descending coronary artery; LCX, left circumflex coronary artery; LDL-C, low density lipoprotein cholesterol; LM, left main coronary artery; LVEF, left ventricular ejection fraction; MI, myocardial infarction; NSTEMI, non ST segment elevation myocardial infarction; PCI, percutaneous coronary intervention; RCA, right coronary artery; STEMI, ST segment elevation myocardial infarction.

### Twelve-months clinical outcomes

**Figure 2:**
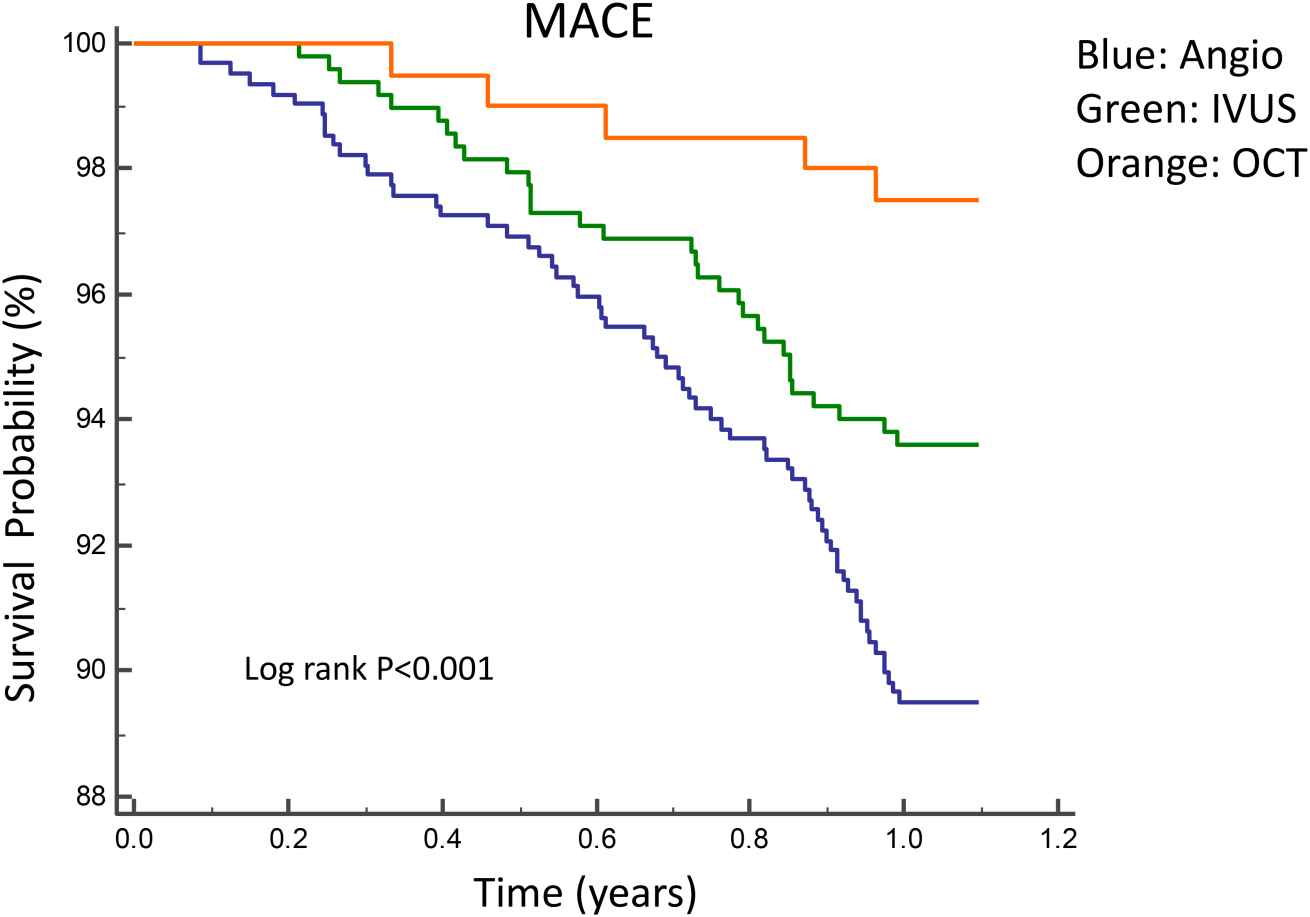
Kaplan–Meier curves for 1-year MACE among angiography-guided, IVUS-guided and OCT-guided PCI. MACE, Major adverse cardiac events.

**Figure 3:**
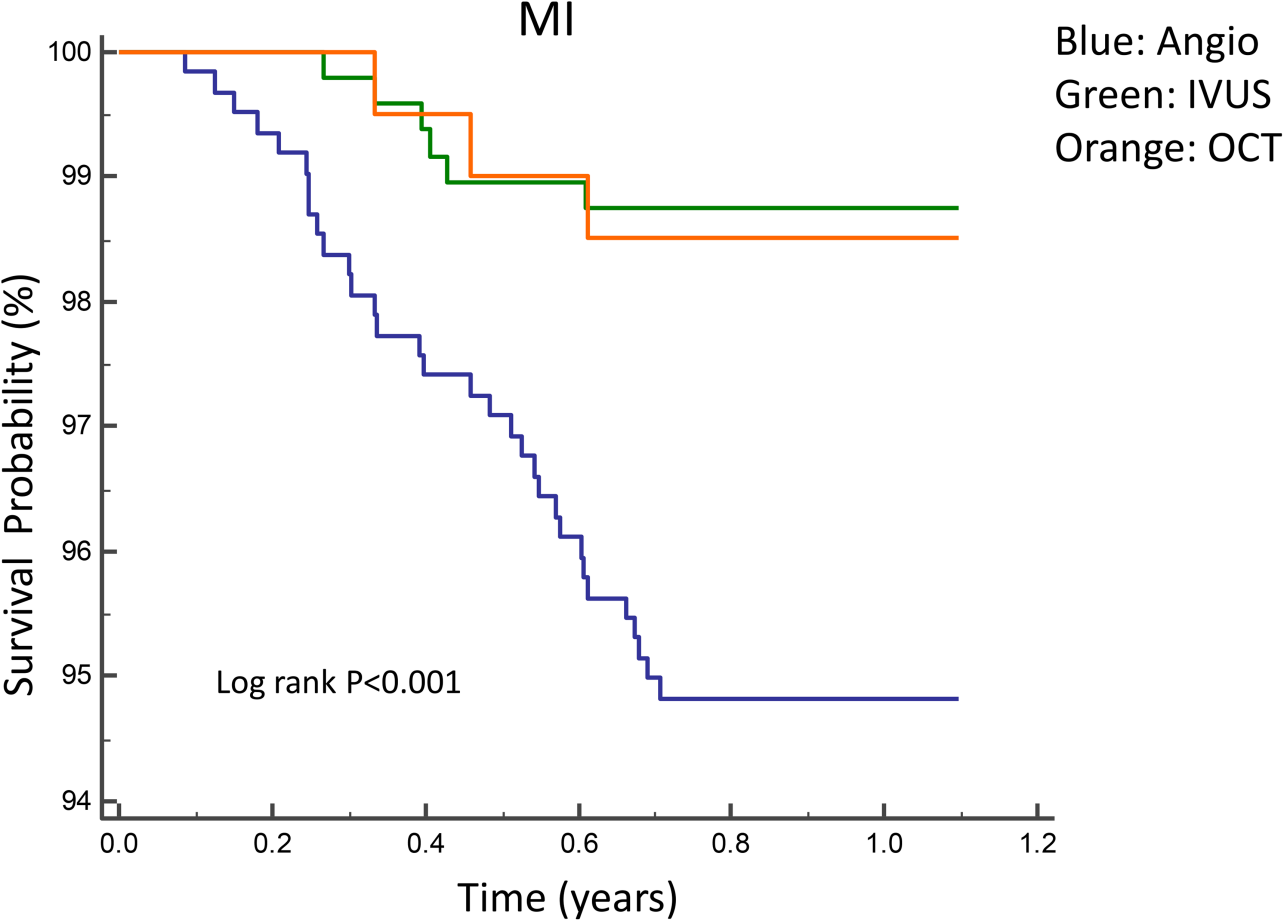
Kaplan–Meier curves for 1-year MI among angiography-guided, IVUS-guided and OCT-guided PCI.

Figures 2, 3 illustrate the comparisons of clinical outcomes among angiography-guided, IVUS-guided, and OCT-guided PCI groups. Kaplan–Meier curves revealed significant differences in the risk of MI and MACE across the three groups, with the OCT-guided group exhibiting superior clinical outcomes. At 12 months, MI and MACE rates were significantly lower in the intravascular imaging-guided PCI group (MI: 1.3%, *P* = 0.001; MACE: 5.2%, *P* = 0.036, respectively; Table 5). The PSM analysis confirmed the continued significance of lower MACE rates (MACE: 1.7%, *P* = 0.028; Table 6).

**Table 5:** 1-year clinical outcomes between 2 groups of patients with acute myocardial infarction.

Variables	Angiography Alone	Intra-Vascular Imaging	*P*-value
	*N* = 620	*N* = 684	
CV death	28 (4.5%)	33 (4.8%)	0.762
Non fatal MI	32 (5.2%)	9 (1.3%)	0.001
TVR	12 (1.9%)	10 (1.5%)	0.422
MACE	65 (10.5%)	36 (5.2%)	0.036

CV, cardiovascular; MACE, major adverse cardiovascular events; MI, myocardial infarction; TVR, target vessel revascularization.

**Table 6:** Propensity score matching of the impact of imaging modalities guided PCI on 1-year clinical outcomes.

Variables	Angiography alone	Intra-Vascular Imaging	*P*-value
	*N* = 598	*N* = 598	
CV death	15 (2.5%)	20 (3.3%)	0.561
Non fatal MI	14 (2.3%)	3 (0.5%)	0.081
TVR	10 (1.7%)	2 (0.3%)	0.156
MACE	25 (4.2%)	10 (1.7%)	0.028

CV, cardiovascular; MACE, major adverse cardiovascular events; MI, myocardial infarction; TVR, target vessel revascularization.

Subgroup analysis revealed that the 12-month CV death and MACE rates were significantly lower in the OCT-guided PCI group (MACE: 2.5%, *P* = 0.032; CV death: 1.5%, *P* = 0.016, respectively; Table 7). The PSM analysis confirmed the significance of the reduced 12-month MACE and CV death rate (MACE: 2.6%, *P* = 0.014; CV death: 1.6%, *P* = 0.032, respectively; Table 8).

**Table 7:** One-year clinical outcomes of IVUS-guided vs. OCT-guided PCI.

Variables	IVUS	OCT	*P*-value
	*N* = 483	*N* = 201	
CV death	30 (6.2%)	3 (1.5%)	0.016
Non fatal MI	6 (1.2%)	3 (1.5%)	0.872
TVR	8 (1.7%)	2 (0.9%)	0.255
MACE	31 (6.4%)	5 (2.5%)	0.032

CV, cardiovascular; MACE, major adverse cardiovascular events; MI, myocardial infarction; TVR, target vessel revascularization.

**Table 8:** Propensity score matching of the impact of different imaging modalities guided PCI on 1-year clinical outcomes.

Variables	IVUS	OCT	*P*-value
	*N* = 190	*N* = 190	
CV death	10 (5.3%)	3 (1.6%)	0.032
Non fatal MI	4 (2.1%)	2 (1.1%)	0.561
TVR	6 (3.2%)	1 (0.5%)	0.081
MACE	20 (10.5%)	5 (2.6%)	0.014

CV, cardiovascular; MACE, major adverse cardiovascular events; MI, myocardial infarction; TVR, target vessel revascularization.

### Predictors of 1-year MACE

Cox regression analysis revealed the use of intravascular imaging as a strong predictor of improved 1-year MACE outcomes [Hazard Ratio (HR) = 0.38, *P* < 0.001; Table 9]. When adjusted for age, sex, diabetes, and hypertension in multivariate analysis, intravascular imaging use remained a robust predictor of favorable 1-year MACE results (HR: 0.29, *P* = 0.001; Table 9). Additionally, DM significantly predicted poorer 1-year MACE outcomes (HR = 3.28, *P* = 0.001; Table 9).

**Table 9:** COX regression analysis for 1-year MACE in two groups (angiography-alone vs. intravascular guided).

Variables	Uni-variate analysis hazard ratio (95% CI)	*P*-value	Multi-variate analysis hazard ratio (95% CI)	*P*-value
Age	1.12 (0.96, 1.21)	0.111	1.14 (0.96, 1.28)	0.138
Sex	0.76 (0.04, 2.16)	0.211	0.59 (0.21, 1.89)	0.261
Diabetes	4.16 (2.12, 8.41)	<0.001	3.28 (1.63, 6.55)	0.001
Hypertension	1.66 (1.02, 2.58)	0.026	1.42 (0.88, 2.37)	0.138
Intra-vascular imaging guided PCI	0.38 (0.15, 0.66)	<0.001	0.29 (0.12, 0.56)	0.001

PCI, percutaneous coronary intervention.

## Discussion

### Main findings and clinical relevance

This study demonstrated that intravascular-guided PCI is associated with improved 1-year non fatal MI and MACE outcomes compared to that associated with angiography-guided PCI alone. These findings are particularly relevant in the context of acute myocardial infarction (AMI) where optimizing stent deployment is critical. Furthermore, intravascular imaging serves as a predictor of enhanced 1-year MACE outcomes after adjusting for various variables. Moreover, subgroup analysis revealed that OCT use is linked with reduced 1-year CV death and MACE in AMI PCI.

### Role of IVUS guidance in AMI patients

Theoretically, the advantage of IVUS guidance in AMI PCI may relate to improved stent implantation guidance, a known predictor of reduced restenosis or ST in elective PCIs. However, in AMI patients, determining the appropriate stent size, length, and optimization of stent deployment can be challenging due to the presence of a large thrombus burden, which may result in under- or oversized stent selection, smaller minimal stent CSA from under-expansion, or perforation from overexpansion. Large observational cohort studies, randomized trials, and meta-analyses have demonstrated a lower incidence of TVR and fewer MACEs, MI, and ST in IVUS-guided elective PCI compared to those associated with angiography-guided intervention (22–28). The HORIZONS-AMI trial's IVUS sub-study indicated that the final post-procedure minimal stent cross-sectional area (CSA) was a strong predictor of early ST and in-stent restenosis in AMI patients. A well-expanded stent with a final stent CSA ≥5 mm^2^ by IVUS was an independent predictor of freedom from ST and restenosis in AMI PCI (29). Furthermore, the ADAPT-DES study observed that IVUS-guided AMI PCI was associated with lower 1-year rates of ST, MI, and MACE (17). However, the benefit of routine IVUS guidance in AMI PCI remains controversial. A Korean study reported no prevention of ST events with IVUS-guided STEMI PCI, which was associated with a higher adverse event rate. This study noted greater stent lengths and a higher number of stents used in the IVUS-guided arm, potentially negating the benefits of IVUS for patients with stable CAD undergoing elective PCI (18). The CREDO-Kyoto AMI registry sub-analysis (19) reported no benefit of routine IVUS guidance in reducing TVR, ST, and mortality in AMI patients undergoing PCI. Conversely, a 2022 meta-analysis found that IVUS-guided AMI PCI was associated with a lower risk of all-cause mortality, MACE, and TVR compared to those associated with angio-guided PCI (30). This analysis included nine studies with a total of 838,902 patients and a maximum follow-up of five years, potentially supporting the use of IVUS in AMI PCI. However, data comparing IVUS-guided and OCT-guided AMI PCI remain limited. Our study observed that intravascular image-guided PCI was associated with lower event rates compared to that associated with angiography-guided PCI. Moreover, OCT-guided PCI was linked to even lower event rates than that associated with IVUS-guided PCI.

### Role of OCT guidance in AMI patients

IVUS has fundamental limitations, such as slower catheter pullback, poor axial resolution (100–200 µm), and limited discrimination of plaque subtypes compared to those associated with OCT. OCT provides superior resolution (10 µm) images, capable of more accurately identifying lesion characteristics, dissection, plaque prolapse, stent mal-apposition, and strut coverage compared to that associated with IVUS (6–8). However, OCT is unsuitable for investigating large and totally occluded vessels, coronary arteries with massive dissection, left main or RCA ostial lesion and periprocedural stent-related complications. The CLI-OPCI registry results suggested that OCT use in patients undergoing PCI could improve clinical outcomes (31). In the CLI-OPCI study, OCT guidance was associated with a significantly lower risk of cardiac death and MI, even after multivariable analysis (OR = 0.49, *P* = 0.037) or propensity-score-adjusted analyses. The CLI-OPCI II study demonstrated that an in-stent minimum lumen area >4.5 mm^2^ [hazards ratio (HR): 1.64, *P* = 0.040] was an independent predictor of better clinical outcomes in non-left main lesion (32). A multicenter RCT, the OCTOBER trial, published in 2023, reported that OCT-guided PCI is associated with significantly lower MACE at 2 years compared to that associated with angio-guided PCI in complex bifurcation lesions (HR = 0.7, *P* = 0.035) (33). The cardiovascular outcomes were not affected by the increased contrast use and longer procedure time (33). Another meta-analysis supported this finding and further suggested that OCT use may improve outcomes in AMI patients (34). In OCT-guided AMI PCI, OCT imaging can identify the location of the culprit lesion, the site of thrombosis, and the longitudinal extent of disease in the culprit vessel. OCT imaging in AMI culprit lesions can provide useful information to distinguish between stent under-expansion and/or tissue prolapse. Due to its high resolution, OCT is more sensitive to the detection of stent mal-apposition, dissection, thrombus, and tissue protrusion, which may not be identified on angiography alone or even with IVUS. The DOCTORS trial (35) was the first randomized, prospective, multicenter trial to investigate the use of OCT in NSTEMI patients, revealing that OCT findings led to a change in procedural strategy in 50% of the patients, mainly driven by the optimization of stent expansion, and were associated with higher fractional flow reserve (FFR) values at the end of the procedure than those associated with angiography-guided PCI alone. In the present study, OCT-guided AMI PCI was associated with better clinical outcomes. After adjusting for differences in baseline characteristics across groups with different imaging modalities, 1-year MACE remained lowest with OCT guidance in AMI PCI compared to that associated with angiography-guided alone PCI and IVUS-guided PCI. The possible mechanism by which OCT-guided AMI PCI achieves better long-term outcomes may be due to several factors. First, OCT can detect plaque characteristics in more detail. Second, OCT offers superior resolution for thrombus recognition, making it more sensitive in identifying culprit vessels even after spontaneous thrombolysis and reperfusion, particularly in patients with multivessel NSTEMI. Third, the high resolution of OCT allows for more precise detection of stent malapposition, resulting in better stent apposition rates and larger post-stent minimal lumen areas.

### Limitations

This study had several limitations. First, as a single-center study with high-volume OCT and IVUS usage, the results may not be generalizable to centers that do not routinely use invasive coronary imaging. Second, the decision to use IVUS or OCT imaging for PCI guidance, as well as the responses to IVUS and OCT findings, was at the operator's discretion. This could potentially introduce significant selection bias due to factors such as angiographic complexity, diagnostic uncertainty, the patient's hemodynamic instability and time pressure, or the operator's preference for one imaging modality over the other. However, despite some degree of selection bias, the study results enhance the potential that using intravascular imaging, especially OCT, may offer benefits for clinical outcomes. Third, our results may not be applicable to bioresorbable scaffold (BVS) implantation in AMI PCI, as only DES and BMS were used in this study. Fourth, with only 1-year follow-up data available, a larger-scale study with long-term outcomes (beyond one year) is necessary to confirm the superior benefits of OCT guidance observed in this study. Fifth, OCT's inability to image ostial and left main lesions represents another limitation.

While we adjusted for known variables such as age, gender, diabetes mellitus (DM), chronic kidney disease (CKD)/end-stage renal disease (ESRD), and congestive heart failure (CHF) using propensity score matching, unmeasured confounders may still have influenced our results. Factors such as operator experience, patient adherence to medication, and lifestyle changes post-PCI could not be fully accounted for and may have impacted clinical outcomes. As a retrospective study, our findings are subject to inherent biases associated with this study design. These include recall bias, selection bias, and the potential for incomplete or inaccurate data recording. While we utilized robust statistical methods to mitigate these biases, prospective, randomized controlled trials are necessary to validate our findings. In light of these limitations, our study should be interpreted with caution. We emphasize the need for further research, including large-scale, multicenter, prospective studies with long-term follow-up and economic evaluations, to confirm the clinical benefits and cost-effectiveness of OCT and IVUS-guided PCI in AMI patients.

## Conclusion

Angiography remains the standard for guiding procedural decisions during primary PCI; however, it has well-known limitations in providing detailed information on vessel walls and atherosclerotic plaque characteristics. Our study suggests that imaging-guided PCI, particularly with OCT, is associated with improved 1-year clinical outcomes compared to angiography-guided PCI alone. Specifically, OCT-guided PCI in AMI appears to be associated with lower 1-year MACE rates compared to IVUS-guided PCI.

Given the study's limitations, including its single-center design and the focus on short-term outcomes, it is important to recommend OCT as a potential option rather than a universal standard for all PCI in AMI. OCT's superior resolution allows for better characterization of unstable plaques, assessment of thrombus burden, and improved stent apposition, which may be particularly beneficial in complex cases. However, the choice of imaging modality should be individualized based on patient characteristics, procedural context, and available resources. Further research and validation are needed to fully establish the role of OCT in routine clinical practice and to guide more nuanced recommendations.

## Data Availability

The original contributions presented in the study are included in the article/Supplementary Material, further inquiries can be directed to the corresponding authors.
